# Co-Loaded PEGylated Nanoliposomes of Bendamustine and Rutin: Formulation, Release Kinetics, and a Hybrid Predictive Modeling Framework

**DOI:** 10.3390/pharmaceutics18060689

**Published:** 2026-05-31

**Authors:** Ali Al-Samydai, Ali Olamat, Arwa Al Khatib, Jamal Al Nabulsi, Hamdi Al Nsairat, Walhan Alshaer, Sara Al Mahamid, Alaa Alsanabrah, Ahmed S. A. Ali Agha, Hamza AbuOwida

**Affiliations:** 1 Department of Pharmacy, Pharmacological and Diagnostic Research Centre, Al-Ahliyya Amman University, Amman 19111, Jordan; a.alkhatib@ammanu.edu.jo (A.A.K.); h.alnseirat@ammanu.edu.jo (H.A.N.); a.sanabrah@ammanu.edu.jo (A.A.); 2 Department of Medical Engineering, Faculty of Engineering, Al-Ahliyya Amman University, Amman 19328, Jordan; a.olamat@ammanu.edu.jo (A.O.); j.nabulsi@ammanu.edu.jo (J.A.N.); h.abuowida@ammanu.edu.jo (H.A.); 3 Cell Therapy Centre, The University of Jordan, Amman 11942, Jordan; walhan.alshaer@ju.edu.jo; 4 Strathclyde Institute of Pharmacy & Biomedical Sciences, University of Strathclyde, Glasgow G4 0RE, UK; sara.al-mahamid@strath.ac.uk; 5 Department of Pharmaceutical Sciences, School of Pharmacy, The University of Jordan, Amman 11942, Jordan; ahm9220505@ju.edu.jo

**Keywords:** nanoliposomes, drug co-delivery, bendamustine, rutin, drug release kinetics, Weibull modeling, machine learning, predictive modeling, data augmentation, hybrid modeling

## Abstract

**Objectives:** Current liposomal drug delivery studies remain largely formulation-specific and descriptive, with limited predictive capability. This study aimed to develop co-loaded nanoliposomes and establish an integrated framework for predictive analysis of drug release. **Methods:** PEGylated nanoliposomes co-loaded with bendamustine and rutin were prepared using the thin-film hydration method. Physicochemical properties, encapsulation efficiency, and in vitro release were evaluated. An integrated analytical approach combining data augmentation, monotonicity-constrained denoising, Weibull kinetic modeling, and machine learning was applied to characterize and predict release behavior. **Results:** Co-loaded formulations exhibited higher encapsulation efficiency (up to 77.75%) and distinct release profiles compared to single-drug systems. Weibull modeling adequately described nonlinear release kinetics (R^2^ ≈ 0.90–0.94). Machine learning enabled within-formulation prediction of later-stage release from early time points (R^2^ > 0.98; MAE ≈ 0.83–1.00%), although leave-one-formulation-out cross-validation confirmed that cross-formulation generalization remains limited. Reconstructed release curves captured overall formulation-dependent trends, despite variable accuracy in individual kinetic parameters. **Conclusions:** The proposed hybrid framework enables early prediction of drug release and reveals that curve-level behavior may be approximated without precise parameter estimation, though this reflects parameter compensability rather than robust prediction. This work provides a proof-of-concept framework for analyzing nanoliposomal drug delivery systems.

## 1. Introduction

Liposomal nanocarriers represent one of the most clinically advanced drug delivery platforms, offering enhanced pharmacokinetic control, improved stability, and reduced systemic toxicity through tunable lipid bilayer composition and surface engineering [1,2]. Recent studies highlight that lipid composition, cholesterol content, and PEGylation critically regulate membrane rigidity, drug retention, and circulation time, thereby directly shaping release behavior and therapeutic performance [3,4]. Despite these advances, the rational design of liposomal systems remains constrained by the complex and often formulation-specific interplay between physicochemical properties and drug release kinetics.

Co-delivery strategies have increasingly been explored as a rational approach to improve therapeutic outcomes by enabling the simultaneous delivery of multiple agents within a single nanocarrier [5,6,7]. In particular, combining cytotoxic drugs with bioactive phytochemicals has gained considerable attention due to their synergistic modulation of oxidative stress, inflammatory signaling, and resistance-associated pathways, thereby enhancing therapeutic efficacy while potentially reducing adverse effects [8]. Bendamustine, a clinically used alkylating agent, exhibits potent antitumor activity but is associated with dose-limiting toxicities and pharmacokinetic limitations [9]. Rutin, a flavonoid with antioxidant and anti-inflammatory properties, shows therapeutic potential but suffers from poor solubility and low bioavailability, which can be partially overcome through nanocarrier-based delivery systems [10]. However, co-encapsulation of such structurally and physicochemically distinct molecules introduces additional complexity, particularly in terms of encapsulation efficiency and coordinated release behavior.

Drug release from liposomal systems is inherently nonlinear and governed by multiple overlapping mechanisms, including diffusion, bilayer permeability, and drug–lipid interactions [11]. Classical kinetic models such as Higuchi and Korsmeyer–Peppas provide useful empirical descriptions but are often insufficient to capture the full variability of formulation-dependent release profiles [12]. The Weibull model has therefore been increasingly adopted due to its flexibility in describing diverse release kinetics, although it remains primarily descriptive rather than predictive [13].

Concurrently, recent advances in machine learning have enabled predictive modeling of drug release and formulation behavior, offering the potential to reduce experimental burden and accelerate formulation optimization. However, current applications remain limited by small dataset sizes, lack of physically constrained modeling, and insufficient integration with established kinetic frameworks [14,15]. In particular, the combined use of data augmentation, constraint-aware denoising, and hybrid mechanistic–machine learning approaches remains underexplored in nanoliposomal systems.

In this context, the present study introduces a dual-level contribution. First, it develops and characterizes PEGylated nanoliposomes co-loaded with bendamustine and rutin, systematically comparing single- and dual-drug systems in terms of encapsulation efficiency and release behavior. Second, and more importantly, it establishes an integrated analytical framework that combines data augmentation, monotonicity-constrained denoising, Weibull-based kinetic modeling, and machine learning-driven prediction to enable early-to-late release forecasting and kinetic parameter estimation.

This combined experimental–computational strategy extends beyond conventional liposomal characterization by shifting from descriptive analysis toward predictive formulation science. By embedding physical constraints and mechanistic modeling within a data-driven framework, this work aims to provide a scalable approach for interpreting and forecasting release behavior in complex nanoliposomal systems, thereby offering methodological advancement with direct relevance to rational nanocarrier design.

## 2. Materials and Methods

### 2.1. Preparation of Bendamustine and Rutin Nanoliposomes

Dual-loaded nanoliposomes containing bendamustine (Carbosynth, Compton, UK/New Delhi, India) and rutin (Sygnus Biotech, Tokyo, Japan) were prepared using the thin-film hydration method according to the previously reported protocols [16,17,18,19,20,21]. The lipid bilayer consisted of distearoylphosphatidylcholine (DSPC), cholesterol, and DSPE-PEG (2000) amine (Avanti Polar Lipids, Alabaster, AL, USA) at a molar ratio of 65:30:5, with a total lipid concentration of 0.51 mmol.

Briefly, lipids were dissolved in an appropriate organic solvent and evaporated under reduced pressure using a rotary evaporator to form a thin lipid film on the inner surface of a round-bottom flask. The dried lipid film was hydrated with an aqueous solution containing bendamustine and rutin (each at 2 mg/mL) under controlled temperature and gentle stirring to facilitate vesicle formation.

The resulting multilamellar vesicles were further processed by probe sonication or membrane extrusion to reduce particle size and obtain nanosized liposomes with improved size uniformity. The incorporation of DSPE-PEG (2000) amine was intended to enhance colloidal stability, reduce aggregation, and provide steric stabilization.

This formulation strategy enabled the preparation of single-drug (B, R) and dual-drug (BR, RB) nanoliposomes for subsequent physicochemical characterization and release analysis. The dual-drug formulations are distinguished by the primary drug of interest and the relative loading priority; BR denotes Bendamustine-Rutin co-loaded nanoliposomes, while RB denotes Rutin-Bendamustine co-loaded nanoliposomes.

### 2.2. Dataset and Variables

The dataset comprised four nanoliposomal formulations evaluated for two primary outcomes: encapsulation efficiency (EE, %) and in vitro cumulative drug release (%). The formulations included bendamustine-loaded nanoliposomes (B), rutin-loaded nanoliposomes (R), bendamustine-priority co-loaded nanoliposomes (BR), and rutin-priority co-loaded nanoliposomes (RB) Encapsulation efficiency values were reported as mean ± standard deviation (SD). Drug release profiles were measured at eight time points (0.5, 1, 2, 4, 6, 24, 48, and 72 h). Physicochemical characterization data, including particle size, polydispersity index (PDI), zeta potential, and high-performance liquid chromatography (HPLC) quantification of drug loading, were available for all formulations and incorporated into the analysis where relevant.

### 2.3. Physicochemical Characterization

Physicochemical characterization of the nanoliposomal formulations was performed to evaluate particle size distribution, polydispersity index (PDI), and surface charge.

The average particle size (Z-average), PDI, and zeta potential of the bendamustine-loaded (B), rutin-loaded (R), co-loaded bendamustine–rutin (BR), and blank formulations were measured using dynamic light scattering (DLS) with a Zetasizer (Malvern Instruments Ltd., Malvern, UK). Prior to analysis, samples were diluted (1:20) with distilled water to minimize multiple scattering effects.

Particle size (Z-average diameter) and PDI were measured at 25 °C using disposable sizing cuvettes, and results were reported as intensity-weighted distributions. Zeta potential measurements were carried out using electrophoretic light scattering with disposable zeta cells, and values were calculated based on the Smoluchowski approximation.

To assess physicochemical stability, nanoliposomal formulations were stored at 2 °C, and measurements were performed at predefined time intervals (0, 1, 2, 3, 7, 14, and 30 days). All measurements were conducted in triplicate, and results were expressed as mean ± standard deviation.

### 2.4. Structure and Morphology of Nanoliposomes

The morphological characteristics of the bendamustine–rutin co-loaded nanoliposomes were evaluated using Scanning Transmission Electron Microscopy (STEM) (SPI supplies, West Chester, USA). The BR formulation was selected as the representative model for morphological analysis due to its superior encapsulation efficiency (77.75%), optimal compact size (99.71 nm), and enhanced long-term physicochemical stability compared to the single-drug and RB formulations. A drop of the diluted nanoliposomal suspension was applied to a carbon-coated copper grid and allowed to air-dry at room temperature, based on the protocol of Alrubaye et al. (2025) [6]. The samples were then examined using Versa 3D STEM (FEI, Eindhoven, The Netherlands) in Bright Field (BF) mode at an accelerating voltage of 30.00 kV and a beam current of 31 pA. Micrographs were captured at a magnification of 80,000× with a working distance of 10.6 mm. The particle size and distribution were analyzed using integrated imaging software, with measurements calibrated against a 500 nm scale bar to ensure accurate, reproducible results.

### 2.5. HPLC Analysis of Drug Loading and Encapsulation

Quantification of bendamustine and rutin, as well as determination of encapsulation efficiency, were performed using high-performance liquid chromatography (HPLC) (Shimadzu Corp., Kyoto, Japan).

Chromatographic analysis was carried out using a C18 reversed-phase column (Universil HS C18, 5 µm, 250 mm length). The column temperature was maintained at 40 °C.

The mobile phase consisted of methanol (A) and normal saline (B) in a 50:50 (*v*/*v*) ratio. The flow rate was set at 1.0 mL/min and the injection volume was 10 µL. Detection was performed at 350 nm, with a total run time of 7 min.

Retention times were approximately 2.29 min for rutin and 4.0 min for bendamustine under the optimized conditions.

Stock solutions were prepared by dissolving rutin in methanol and bendamustine in normal saline. Calibration standards and working solutions were prepared by appropriate dilution using the mobile phase. The analytical method was validated over a concentration range of 0.05–1 mg/mL for both compounds.

### 2.6. Encapsulation Efficiency Determination

Encapsulation efficiency (EE) was determined using HPLC-UV detector (Shimadzu Corp., Kyoto, Japan) analysis following separation of free drug from liposomal formulations.

The method was adapted from a previously reported protocol [2], with compound-specific optimization of detection parameters, including adjustment of mobile phase composition and detection conditions for each analyte. A unified sample preparation protocol was applied for all formulations. Encapsulation efficiency (EE%) was calculated as:

$$EE(\%)=\frac{\mathrm{Encapsulated}\;\mathrm{drug}}{\mathrm{Total}\;\mathrm{drug}\;\mathrm{added}}\times 100$$

All measurements were performed in triplicate and reported as mean ± SD.

### 2.7. In Vitro Release Assessment

In vitro cumulative drug release was assessed for all four nanoliposomal formulations at predefined time points (0.5, 1, 2, 4, 6, 24, 48, and 72 h) using the dialysis bag diffusion technique. Briefly, 2 mL of each nanoliposomal suspension was enclosed in a cellulose ester dialysis membrane (molecular weight cut-off: 12,000–14,000 Da). The sealed dialysis bags were immersed in 50 mL of phosphate-buffered saline (PBS, pH 7.4) and incubated at 37 ± 0.5 °C in a shaking water bath at 100 rpm to maintain sink conditions and ensure uniform dispersion.

At each sampling point, 1 mL of the external release medium was withdrawn and immediately replaced with an equal volume of fresh, prewarmed PBS to maintain a constant volume. The concentration of the released drug was determined using HPLC analysis, as described in [7,22].

### 2.8. Data Augmentation Strategy

**Figure 1:**
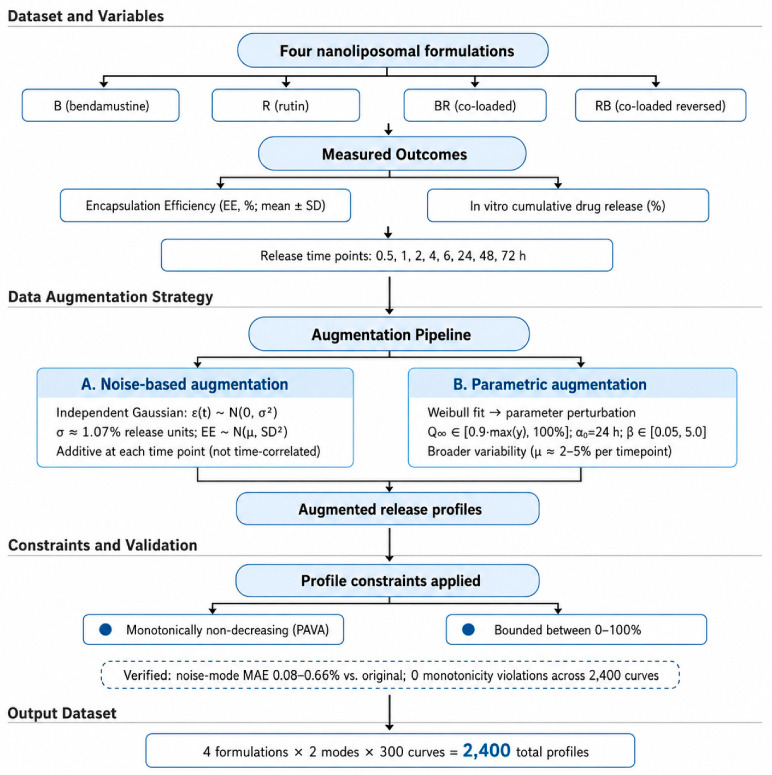
Analysis workflow for the four-formulation nanoliposome dataset, including augmentation, denoising, kinetic modeling, and early-to-late forecasting.

To enhance data robustness and enable uncertainty quantification, a data augmentation strategy was employed to generate replicate-like release profiles as demonstrated in Figure 1.

To enhance data robustness and enable uncertainty quantification, a data augmentation strategy was employed to generate replicate-like release profiles. For each formulation, two complementary approaches were implemented, yielding 300 augmented curves per formulation per mode (2400 total across 4 formulations × 2 modes).

In the first approach (noise-based augmentation), independent Gaussian perturbations $\varepsilon \left(t_{i}\right)\sim N\left(0,\sigma^{2}\right)$ were applied additively to the observed release value at each time point. The noise amplitude σ was approximately constant across time points, with a grand mean of 1.07% release units across all formulations (formulation-specific values: B = 1.26%, BR = 1.07%, RB = 1.08%, R = 0.87%). The overall mean perturbation was +0.13% (near zero), confirming unbiased noise generation. For each augmented curve, the associated encapsulation efficiency was sampled from a normal distribution matching the experimental statistics:

$$EE\_sample\;\sim \;N(EE\_mean,\;{EE\_SD}^{2}).$$

In the second approach (parametric augmentation), the Weibull release model $Q\left(t\right)=Q\infty \left(1-exp\left(-{\left(t/\alpha \right)}^{\beta }\right)\right)$ was fitted to each formulation’s observed profile using nonlinear least squares (Nelder–Mead simplex, MaxIter = 5000, MaxFunEvals = 20,000). Bounded parameter transforms were applied during optimization: $Q_{\infty }$ was constrained to [0.9 × max(y), 100%] via logistic transform (initial value: 1.2 × max(y)); α was constrained to (0, ∞) via log transform (initial value: 24.0 h); and β was constrained to [0.05, 5.0] via logistic transform (initial value: 0.8). The fitted parameters were then perturbed to generate synthetic release curves, producing broader variability than the noise mode (per-timepoint standard deviations of 2–5% vs. ~1% for noise mode). $Q_{\infty }$ was bounded from below by the observed 72 h release value.

All augmented profiles were constrained to be monotonically non-decreasing and bounded within [0%, 100%] cumulative release. Post hoc verification confirmed zero monotonicity violations across all 2400 curves. The Augmentation_sanity_check reports mean absolute errors between augmented population means and original values of 0.08–0.66% for noise-mode and 0.16–8.59% for parametric-mode, confirming tight agreement and physically plausible variability, respectively.

### 2.9. Monotone Denoising (Isotonic Regression)

Because cumulative drug release is inherently non-decreasing over time, isotonic regression was applied to enforce this physical constraint and reduce experimental noise. For observed release values $y(t_{i})$ at time points $t_{i}$, a monotonic sequence $\hat{y}(t_{i})$ was estimated by minimizing the sum of squared deviations subject to monotonicity constraints using the Equation of:

$$\underset{ŷ}{\min}\sum_{i}{\left(y\left(t_{i}\right)-ŷ\left(t_{i}\right)\right)}^{2}subject\;to\;ŷ\left(t_{1}\right)\leq ŷ\left(t_{2}\right)\leq \cdots \leq ŷ\left(t_{n}\right)$$

The optimization was solved using the pool-adjacent-violators algorithm (PAVA), yielding an efficient, exact solution. The resulting monotone-denoised profiles were subsequently used for downstream kinetic modeling and predictive analyses.

### 2.10. Kinetic Modeling of Release Profiles

Each release profile was characterized using a Weibull model of the form:

$$Q(t)=Q_{\infty }\left(1-exp\left[-{\left(\frac{t}{\alpha }\right)}^{\beta }\right]\right)$$

where Q(t) represents cumulative release (%), $Q_{\infty }$ is the asymptotic release (%), α is a scale parameter (time constant, h), and β is a shape parameter describing the release mechanism.

To minimize over-extrapolation in cases where a plateau was not reached within 72 h, $Q_{\infty }$ was fixed to the observed release value at 72 h for each profile. The parameters α and β were estimated using nonlinear least squares.

The time required to reach 50% of $Q_{\infty }$($t_{50}$) was calculated as:

$$t_{50}=\alpha (ln\;2{)}^{1/\beta }$$

Model performance was evaluated using the coefficient of determination ($R^{2}$).

The half-release time t_50_ was calculated analytically from the fitted Weibull parameters as $t^{50}=\alpha \times {\left(ln2\right)}^{1/\beta },$ representing the time required to reach 50% of the formulation-specific asymptotic release Q∞, not 50% absolute cumulative release.

### 2.11. Early-to-Late Forecasting of Release

To enable prediction of long-term release behavior from early measurements, a supervised learning model was developed. The input features included release values at early time points (0.5, 1, 2, and 4 h), encapsulation efficiency (EE), and a one-hot encoding of formulation identity. The target outputs were cumulative release values at 6, 24, 48, and 72 h.

A multi-output ridge regression model (L2-regularized linear regression) was employed. The dataset was split into training and testing sets using a formulation-stratified 80/20 split to ensure balanced representation. Model performance was evaluated using mean absolute error (MAE), root mean square error (RMSE), and $R^{2}$ for each predicted time point.

### 2.12. Machine Learning-Based Kinetic Parameter Prediction and Curve Reconstruction

To further characterize release kinetics, a regression model was trained to predict Weibull parameters ($Q_{\infty },\alpha ,\beta$) from early-stage data (0.5, 1, 2, and 4 h), along with EE and formulation identity.

The predicted parameters ($Q_{\infty }^{ML},\alpha^{ML},\beta^{ML}$) were subsequently used to reconstruct continuous release profiles using the Weibull equation. Model performance was assessed using MAE and RMSE for both parameter prediction and reconstructed release values across all experimental time points.

Both ridge regression models employed a closed-form solution $W={\left(X^{T}X+\lambda I\right)}^{-1}X^{T}Y$ with the intercept term unpenalized (λ = 1.0). The dataset was split using a formulation-stratified 80/20 holdout with random seed rng (42). Input features comprised release values at 0.5, 1, 2, and 4 h, encapsulation efficiency, and one-hot encoded formulation identity. For Weibull parameter prediction (Section 3.8), target parameters were transformed for stable regression: Q∞ was logit-transformed, while α and β were log-transformed, with predictions back-transformed via inverse logit and exponentiation before curve reconstruction.

## 3. Results and Discussion

The results are presented in two phases: an initial comparative screening of all single-drug (B, R) and dual-drug (BR, RB) systems, followed by an in-depth characterization of the lead formulation. The BR formulation was selected as the primary model for advanced morphological and predictive analysis due to its significantly higher encapsulation efficiency and superior physicochemical stability compared to the RB serie.

### 3.1. Encapsulation Efficiency

Encapsulation efficiency (EE) varied across the four nanoliposomal formulations, indicating formulation-dependent differences in drug loading capacity. The bendamustine–rutin co-loaded formulation (BR) exhibited the highest encapsulation efficiency (77.75 ± 1.18%), followed by the rutin–bendamustine co-loaded formulation (RB) (44.32 ± 1.16%). The rutin-loaded formulation (R) showed moderate encapsulation (35.04 ± 2.31%), whereas the bendamustine-loaded formulation (B) exhibited the lowest encapsulation efficiency (24.41 ± 4.02%).

**Figure 2:**
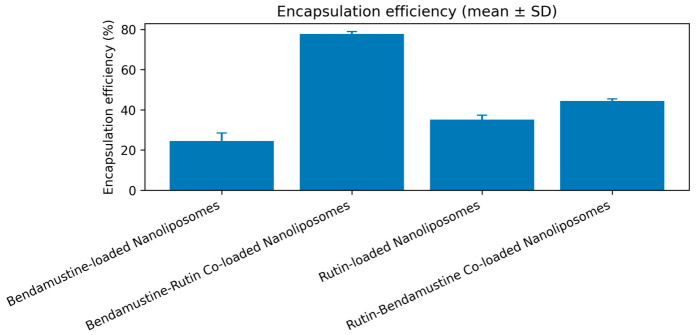
Encapsulation efficiency (mean ± SD) across formulations.

These differences shown in Figure 2 suggest that co-loading of bendamustine and rutin may influence drug–lipid interactions within the nanoliposomal bilayer, potentially enhancing drug retention compared to single-drug systems. The comparatively lower encapsulation efficiency observed for bendamustine alone may reflect differences in physicochemical compatibility with the lipid matrix under the applied formulation conditions.

### 3.2. HPLC Performance and Analytical Suitability

**Figure 3:**
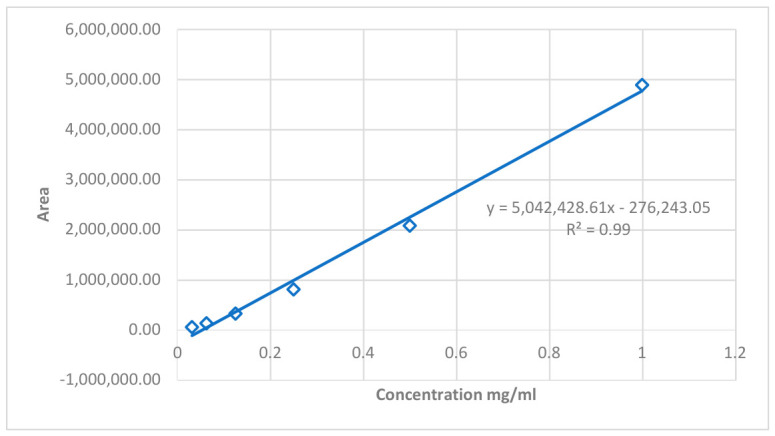

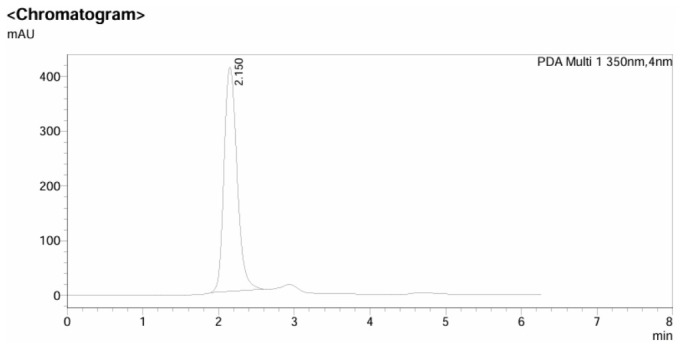
Representative chromatograms of rutin calibration standards (0.03−1 mg/mL), demonstrating a linear increase in peak area with concentration and stable retention time (2.150 min) across injections.

Chromatographic analysis was conducted to evaluate the detection and separation of rutin and bendamustine under different preparation conditions and to support quantitative interpretation of encapsulation efficiency. Calibration data for both analytes showed a consistent increase in peak area with increasing concentration over the investigated range (0.03–1 mg/mL). For rutin, retention time remained stable within a narrow interval of approximately 2.1 min across all calibration standards, with minimal variation observed between injections as shown in Figure 3.

**Figure 4:**
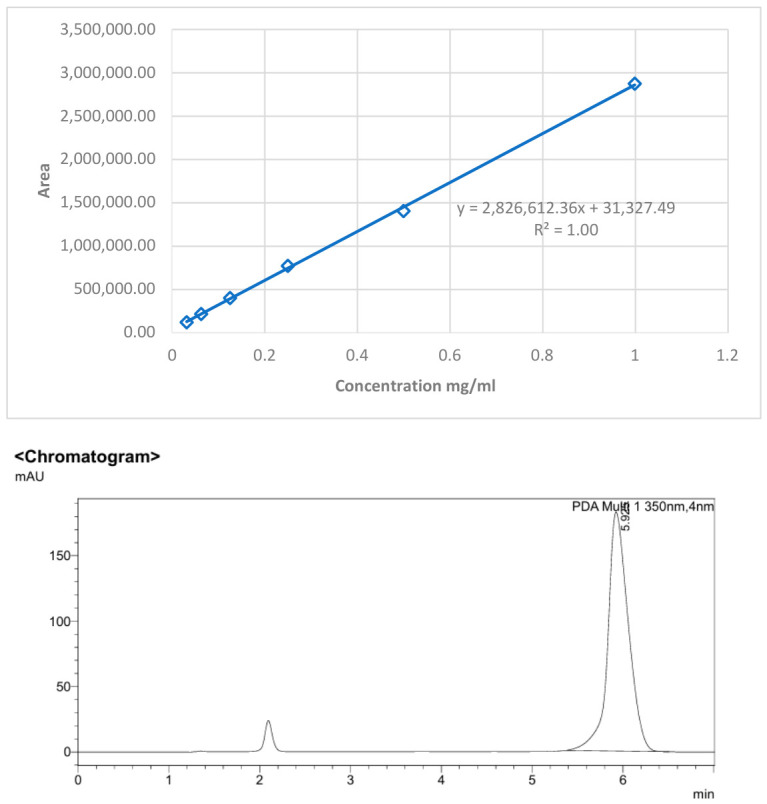
Representative chromatograms of bendamustine calibration standards prepared in normal saline, demonstrating a concentration-dependent increase in peak area and stable retention times (5.95 min).

Bendamustine calibration performed in normal saline exhibited retention times within a relatively confined range of approximately 5.9 min, with peak area increasing consistently as concentration increased, as shown in Figure 4.

These observations indicate a stable detector response within the tested range. Analysis of mixed samples showed two distinct peaks corresponding to rutin and bendamustine, with rutin eluting earlier (~2.1 min) and bendamustine eluting later (~5.9 min). The separation between the two analytes was maintained across all samples, with no evidence of co-elution or significant interference. Retention times and peak profiles were comparable across replicate injections.

A difference in retention behavior was observed for bendamustine between calibration and mixed-sample conditions, where it eluted earlier in mixed samples than in calibration solutions. This suggests that bendamustine retention is influenced by the sample matrix. In contrast, rutin retention remained consistent across conditions. Despite this variation, chromatographic separation between the analytes was preserved. These findings indicate that the method is suitable for qualitative analysis and can support quantitative interpretation when calibration is performed under conditions consistent with the sample matrix.

### 3.3. Physical Characterization

The physical properties of the developed nanoliposomes, including particle size, polydispersity index (PDI), and zeta potential, were thoroughly evaluated. All formulations—blank, rutin-loaded, bendamustine-loaded, and co-loaded—met internationally accepted pharmaceutical standards for nanomedicine. The particle size of all preparations was below 150 nm, with a narrow size distribution around 0.2, confirming their suitability for clinical applications such as parenteral administration.

#### 3.3.1. Particle Size Analysis

One-way ANOVA showed statistically significant differences in particle size among the groups, with an F value of 16.38 and a *p*-value less than 0.001. The co-loaded formulation achieved an optimal mean size of 99.71 ± 4.60 nm, which was significantly smaller than the rutin-loaded group at 118.30 nm. Importantly, the co-loaded formulation maintained a size similar to the blank nanoliposomes at 106.67 nm and the bendamustine-loaded vesicles at 102.45 nm. This indicates that simultaneous drug encapsulation does not increase vesicle size but instead results in a compact and stable nanostructure.

#### 3.3.2. Polydispersity Index and Homogeneity

The PDI values showed significant variation among the formulations, with a *p*-value of 0.045. The co-loaded formulation had a PDI of 0.202 ± 0.005, which remains within the acceptable range for pharmaceutical homogeneity. Although there was a significant difference between the co-loaded formulation and the blank nanoliposomes, which had a PDI of 0.108, no significant differences were observed when compared to the single-drug-loaded formulations. These findings confirm that dual loading preserves nanoparticle uniformity.

#### 3.3.3. Zeta Potential and Surface Charge

All formulations exhibited a negative surface charge, ranging from −12.47 mV to −21.03 mV. As summarized in Table 1, the rutin-loaded and co-loaded formulations exhibited slightly more negative values (−17.53 mV); however, statistical analysis indicated that these differences were not significant, with an F value of 2.565 and a *p*-value of 0.103.

This suggests that the incorporated drugs do not disrupt the electrostatic stability of the liposomal surface.

#### 3.3.4. Chemical Rationale for Successful Co-Loading

The successful co-encapsulation and stability of the formulations can be explained by the strong chemical compatibility between bendamustine and rutin within the lipid bilayer. The liposomal system, composed of DSPC, cholesterol, and DSPE-PEG 2000 amine in a molar ratio of 65:30:5, provides an optimal environment for drug interaction.

Hydrogen Bonding:

Rutin contains multiple phenolic hydroxyl groups that enable hydrogen bonding [21]. These groups interact with the carboxylic acid moiety of bendamustine and the amine and phosphate groups of the lipid headgroups, stabilizing the drugs at the lipid–water interface [23,24].

Pi–Pi Stacking:

The aromatic structures of bendamustine and rutin allow non-covalent stacking interactions [25]. These interactions promote tight molecular packing within the hydrophobic bilayer, supported by cholesterol, which contributes to the compact size of the co-loaded formulation.

Steric and Electrostatic Stabilization:

The presence of DSPE-PEG 2000 amine creates a protective polyethylene glycol layer [26,27]. In addition to hydrogen bonding interactions, this prevents aggregation and maintains nanoparticle stability and uniformity.

#### 3.3.5. STEM Morphological Analysis Results

**Figure 5:**
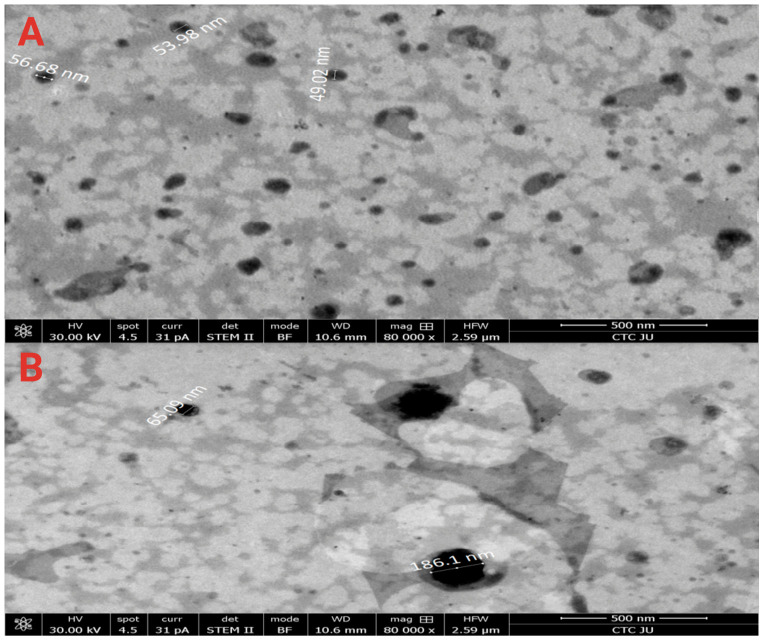
STEM (BF mode) micrographs of the co-loaded (bendamustine–rutin) nanoliposomal formulation at 80,000× magnification. (**A**) Representative image showing uniformly distributed nanoliposomes with diameters in the range of ~49–57 nm. (**B**) Image highlighting the presence of larger vesicular structures and aggregates, with sizes up to ~186 nm. The images confirm predominantly spherical morphology with minimal aggregation.

The morphological evaluation of the co-loaded nanoliposomes was conducted using STEM in Bright Field (BF) mode at an accelerating voltage of 30.00 kV and a magnification of 80,000×. Representative micrographs are presented in Figure 5.

A. Particle Shape and Structure

The nanoliposomes exhibit a predominantly spherical to quasi-spherical morphology. The particles appear as distinct, dark, electron-dense cores, which is characteristic of successful drug encapsulation. The vesicular structure is well-defined, showing minimal signs of aggregation or coalescing in the primary populations, suggesting a stable formulation.

B. Particle Size Distribution

Measurements taken directly from the micrographs reveal a heterogenous size distribution, which can be categorized into two primary observations:

Standard Nanoparticles: As seen in Figure 5A, the majority of the particles are highly uniform with diameters ranging closely between 49 nm and 57 nm (specifically measured at 49.02 nm, 53.98 nm, and 56.68 nm).

Large Vesicles/Aggregates: Figure 5B captures some larger structures, with individual vesicles measured at 65.09 nm and significantly larger complexes reaching 186.1 nm. These larger entities may represent a minor population of multi-lamellar vesicles or localized clusters.

#### 3.3.6. Physicochemical Stability

The physicochemical stability of the nanoliposomal formulations was evaluated based on changes in particle size, polydispersity index (PDI), and zeta potential over time. At initial characterization, all formulations exhibited particle sizes below 300 nm, PDI values below 0.3, and zeta potential values within the range of −30 to +30 mV, which are generally considered acceptable for nanoliposomal systems.

The co-loaded formulation (BR) exhibited the most consistent stability profile, characterized by a gradual increase in particle size (from ~93–104 nm on day 0 to ~135–141 nm on day 30), accompanied by a controlled rise in PDI from ~0.19–0.21 to ~0.24–0.27. Notably, PDI values remained below 0.3 throughout the study, indicating maintenance of a relatively narrow and homogeneous particle size distribution over time. The zeta potential ranged between −12 and −26 mV initially and decreased to approximately −9 mV at later time points. Given the relatively low magnitude of zeta potential values, electrostatic stabilization alone is unlikely to fully account for the observed stability, and the contribution of steric stabilization—particularly from PEG chains—is likely to play a role in maintaining dispersion integrity.

In contrast, the bendamustine-loaded formulation (B) demonstrated pronounced instability, as evidenced by a substantial increase in particle size (~100 nm to ~165–196 nm) and a marked elevation in PDI from ~0.13–0.19 on day 0 to ~0.34–0.37 on day 30. The progressive broadening of PDI reflects increasing heterogeneity within the system. This trend was accompanied by a reduction in zeta potential magnitude to ~−5 to −6 mV, suggesting a limited contribution of electrostatic repulsion and an increased likelihood of particle–particle interactions over time.

The rutin-loaded formulation (R) exhibited intermediate stability, maintaining relatively constant particle size (~118–125 nm) and moderate PDI values (~0.17–0.25) up to day 14. However, on day 30, both particle size (up to ~181 nm) and PDI (up to ~0.50) increased markedly, indicating the development of a broader and more heterogeneous size distribution. This change was associated with a reduction in zeta potential magnitude from ~−21 mV to ~−7 to −9 mV, suggesting a reduced surface charge contribution at later time points.

Similarly, the blank formulation showed relatively stable behavior during the early storage period, with particle size (~106–114 nm) and PDI (~0.04–0.24) remaining within acceptable limits up to day 14. By day 30, an increase in particle size (~136–137 nm) and PDI (~0.25) was observed, alongside a decrease in zeta potential magnitude from ~−12 mV to ~−5 to −6 mV, indicating a gradual reduction in electrostatic contribution to stability.

**Figure 6:**
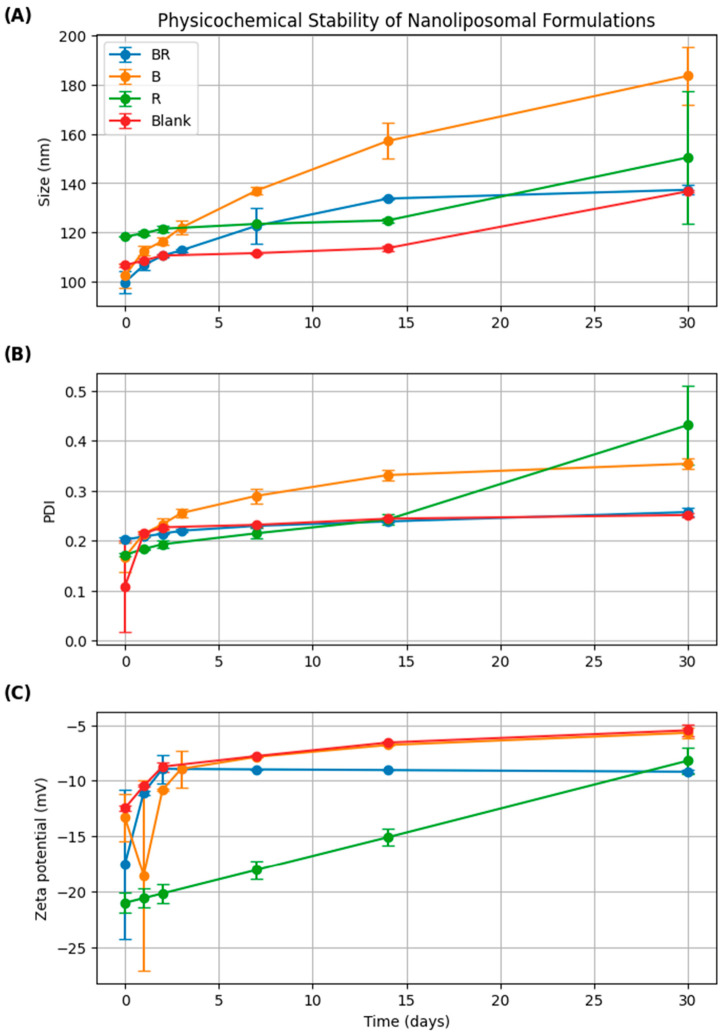
Physicochemical stability of bendamustine-loaded (B), rutin-loaded (R), co-loaded bendamustine–rutin (BR), and blank nanoliposomal formulations over 30 days under storage at 2 °C. (**A**) Particle size (nm), (**B**) polydispersity index (PDI), and (**C**) zeta potential (mV) are presented as a function of time (mean ± standard deviation, SD). All formulations initially exhibited particle sizes below 300 nm and PDI values below 0.3. Over the storage period, a general increase in particle size and PDI was observed, accompanied by a reduction in zeta potential magnitude across all formulations. The BR formulation demonstrated comparatively smaller changes in particle size and PDI over time, indicating a more consistent physicochemical profile relative to the single-drug and blank formulations. Error bars represent standard deviation from replicate measurements.

Across all formulations, an overall increase in particle size and PDI, accompanied by a decrease in zeta potential magnitude, was observed during storage, consistent with progressive changes in colloidal systems over time. Within this context, the co-loaded formulation (BR) exhibited comparatively smaller changes in both particle size and PDI, while maintaining a consistently narrow size distribution throughout the study period. These findings indicate that the BR formulation preserved its physicochemical characteristics more effectively than the single-drug and blank formulations under the same conditions, as shown in Figure 6.

### 3.4. In Vitro Release Behavior

**Figure 7:**
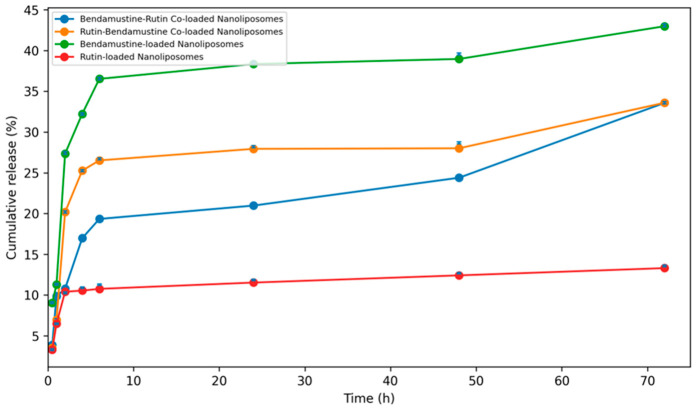
In vitro cumulative release profiles for the four formulations. Error bars indicate 95% CI from replica-like augmentation.

The cumulative drug-release profiles showed distinct patterns across the four formulations. Bendamustine-loaded nanoliposomes showed the highest initial burst release (9.02% at 0.5 h) and the highest cumulative release at 72 h (42.99%), indicating a relatively prolonged release profile under the tested conditions, as shown in Figure 7.

### 3.5. Weibull-Based Kinetic Characterization

Weibull modeling was applied to characterize release kinetics across formulations. The bendamustine–rutin co-loaded formulation (BR) exhibited the highest median time to 50% release (t_50_ ≈ 6.00 h), indicating a slower progression toward its asymptotic release level compared to other formulations, as shown in Table 2.

In contrast, bendamustine-loaded nanoliposomes showed a shorter t_50_ (~1.75 h), consistent with faster release behavior. The Weibull shape parameter (β) varied across formulations, with values generally below or near unity, suggesting non-linear release behavior that may be consistent with diffusion-controlled or complex release mechanisms. The scale parameter (α) also varied between formulations, reflecting differences in release rate. Model fit, assessed using R^2^, ranged from 0.90 to 0.94 across formulations, indicating that the Weibull model provided an adequate description of the release profiles over the observed time range.

### 3.6. Comparison with Classical Release Models

In addition to the Weibull-based analysis, the experimental release profiles were fitted to commonly use kinetic models, including the zero-order, first-order, Higuchi, and Korsmeyer–Peppas equations, to compare their goodness-of-fit across formulations. The comparison, summarized in Table 3, showed that model fit varied according to formulation, indicating that no single classical model described all release profiles equally well.

For the bendamustine-loaded formulation, the Higuchi model gave the highest coefficient of determination ($R^{2}=0.9701$), exceeding the corresponding zero-order, first-order, and Korsmeyer–Peppas fits. This result indicates that the Higuchi equation provided the closest empirical description of release behavior for this formulation within the tested time range. In contrast, for the rutin-loaded, bendamustine–rutin co-loaded, and rutin–bendamustine co-loaded formulations, the Korsmeyer–Peppas model gave the highest $R^{2}$ values (0.9542, 0.9155, and 0.9234, respectively), suggesting that this model better represented the observed release patterns in those systems than the zero-order, first-order, or Higuchi equations.

The Korsmeyer–Peppas release exponent n, estimated from the early release phase (≤6 h), was below 0.5 for all formulations, with values of 0.28 for bendamustine-loaded, 0.35 for rutin–bendamustine co-loaded, 0.31 for bendamustine–rutin co-loaded, and 0.19 for rutin-loaded nanoliposomes. However, the mechanistic thresholds associated with n (e.g., n = 0.5 for Fickian diffusion) were originally derived for slab and cylindrical geometries [28], and for spherical systems the diffusion threshold shifts to n = 0.43. The applicability of these thresholds to nanoliposomal vesicles, where release is governed by bilayer permeability, drug–lipid partitioning, and vesicle destabilization rather than simple matrix diffusion, remains uncertain. The n values reported here are therefore presented as empirical curve-fitting descriptors only and are not interpreted mechanistically. The Weibull model, which makes fewer geometric assumptions, is adopted as the primary kinetic descriptor throughout this work.

Taken together, the model comparison suggests that release behavior was formulation-dependent and that the dominant empirical description varied across systems. The stronger Higuchi fit for the bendamustine-loaded formulation is consistent with a release pattern closely approximated by square-root-of-time dependence, whereas the better Korsmeyer–Peppas fits for the other formulations indicate that a more flexible empirical model was needed to describe early-stage release. These results support the broader conclusion that formulation composition influenced release kinetics and that diffusion-related behavior contributed substantially to drug release across all systems.

### 3.7. Prediction of Later Release from Early Measurements

A ridge regression model was used to predict later-stage release values (6, 24, 48, and 72 h) from early timepoint measurements (0.5–4 h), encapsulation efficiency, and formulation identity. Within the augmented dataset, the model demonstrated consistent within-formulation performance across all evaluated time points, with mean absolute error values in the range of approximately 0.83–1.00% release and coefficient of determination (R^2^) values exceeding 0.98, shown in Table 4.

**Figure 8:**
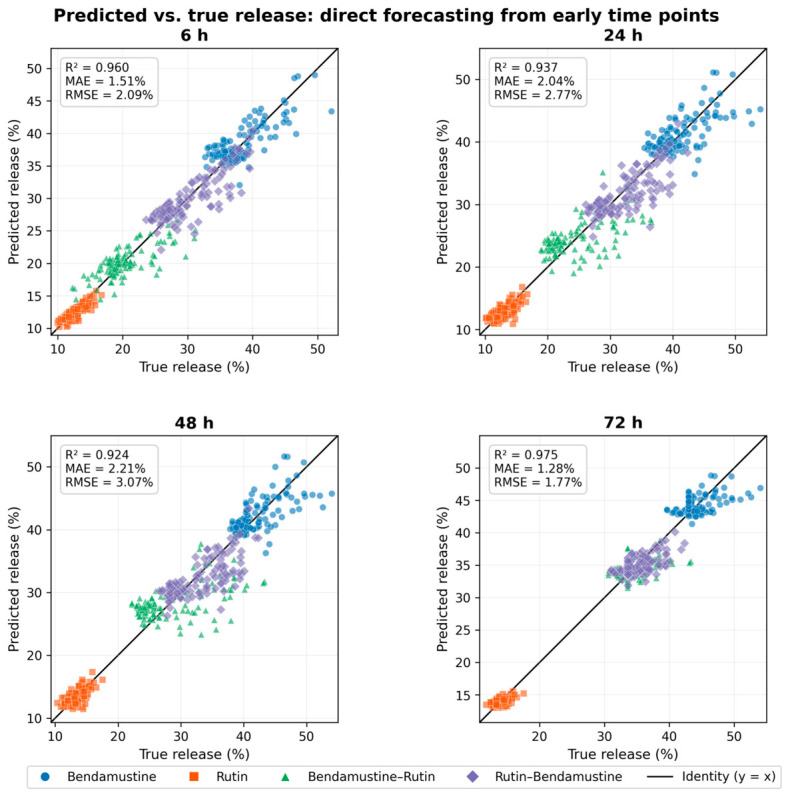
Predicted versus true cumulative release (%) from the direct ridge regression forecasting model (Table 4). Each subpanel corresponds to a specific later-stage time point (6, 24, 48, and 72 h), with predictions generated from early-stage measurements (0.5–4 h), encapsulation efficiency, and formulation identity. Data points represent individual test-set samples (20% stratified holdout), with formulations distinguished by marker shape and color. The solid diagonal line represents perfect agreement (y = x). R^2^, MAE, and RMSE are reported per time point.

Comparison between predicted and observed release values indicated that the model captured the overall trend in release behavior across formulations. Predictions were generally aligned with observed values across the tested range, although some deviations were observed at higher release levels, particularly for later time points. The relationship between predicted and observed release values is shown in Figure 8.

Data points are distributed close to the identity line, indicating agreement between predicted and observed values across all time points. The clustering of points around this line suggests that early-stage release measurements provide sufficient information to estimate later-stage release behavior within the studied system. However, some dispersion is evident, particularly at higher release values, reflecting the increasing uncertainty associated with extrapolation beyond early time points.

Overall, these results indicate that the model provides a reasonable approximation of release behavior based on early measurements. The interpretation of predictive performance should take into account the limited number of original experimental observations and the use of augmented data in model development.

These MAE values reflect direct input–output regression and differ from the Weibull-based reconstruction errors in Section 3.8, where parameter estimation intermediate introduces additional uncertainty.

### 3.8. ML-Based Curve Reconstruction from Predicted Weibull Parameters

In comparison to the direct forecasting technique proposed in Section 3.7, where the measurement values from an early time stage are mapped onto the corresponding release values on a later time stage through a one-step regression process (MAE = 0.83–1.00%, Table 4), the present methodology involves a two-step procedure, wherein the estimation of Weibull parameters occurs based on the early data set, followed by the reconstruction of release curves using the Weibull model. Nonlinear error propagation is associated with this procedure, since inaccuracies in predicting values of α (MAE = 151.54) and Q∞ (MAE = 14.81) are exponentially magnified in the course of calculating release values according to the Weibull function (MAE = 2.98% at 0.5 h to 11.87% at 72 h, Table 6). In practice, the direct technique is more appropriate when exact release values at specific time stages are required.

β was determined with little margin of error (MAE = 0.13), while α showed significantly higher errors (MAE = 151.54) because of the compensable nature of these parameters. Therefore, the use of predictions for Weibull parameters is not advised for any kind of mechanism comparison among various formulae, and it is advisable to use direct predictions (shown in Table 4).

**Figure 9:**
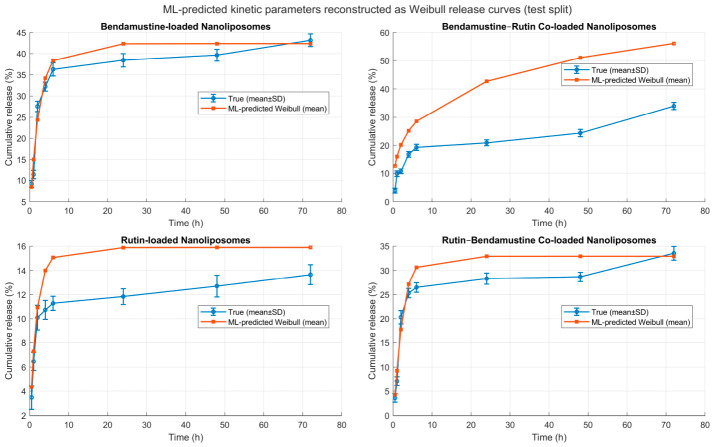
Release curves reconstructed from ML-predicted Weibull parameters on the test set, shown as predicted mean curves relative to the corresponding experimental mean ± SD profiles for each formulation.

Machine learning-based prediction of Weibull kinetic parameters was used to reconstruct release curves from early-stage measurements on the held-out test set. The reconstructed profiles are shown in Figure 9 as the mean predicted Weibull curves relative to the corresponding mean experimental release profiles.

At the formulation level, the reconstructed curves reproduced the overall shape of the release trajectories and preserved the main differences among formulations, including the faster release behavior of the bendamustine-loaded system, the lower cumulative release of the rutin-loaded system, and the intermediate behavior of the co-loaded formulations. This visual agreement indicates that early release measurements contained information relevant to the general form of later-stage release behavior.

At the same time, quantitative evaluation showed that prediction of the Weibull parameters themselves was uneven across parameters. As summarized in Table 5, prediction error was relatively small for the shape parameter β, whereas substantially larger errors were observed for $Q_{\infty }$, α, and the derived $t_{50}$.

These results indicate that reconstruction of the overall curve pattern did not necessarily require accurate recovery of each individual kinetic parameter, and that different combinations of predicted parameters may yield similar release profiles at the curve level.

The large prediction error of α (MAE = 151.54, compared with the actual values between 1.89 and 13.79) suggests parameter compensability, meaning that different α-β combinations will produce almost identical plots in the 0–72 h interval. In this context, it is inappropriate to use individual parameters for explanation purposes or extrapolations outside the observation period.

Curve-level errors are summarized in Table 6. The magnitude of error increased progressively from the earliest time points to later stages of release, with lower MAE and RMSE values at 0.5–1 h and larger errors at 24–72 h.

**Table 6:** Curve-level prediction error (MAE, RMSE) at each timepoint for release curves reconstructed from ML-predicted Weibull parameters.

Time_h	MAE	RMSE
0.5	2.984567	4.620607
1	3.582633	4.45787
2	5.015717	6.299752
4	6.697853	7.829849
6	7.899591	9.272635
24	12.2454	14.80718
48	13.33884	16.56489
72	11.8706	14.49873

This pattern suggests that uncertainty accumulated as the reconstructed curves extended further beyond the early measurements used as model inputs. Nevertheless, the reconstructed curves remained qualitatively consistent with the observed formulation-specific release behavior, particularly with respect to rank ordering and general trajectory.

Taken together, these findings indicate that ML-based reconstruction was more informative for approximating overall release profiles than for precise estimation of individual Weibull parameters. The approach appears useful for capturing broad formulation-dependent release patterns from early data, but the relatively large errors associated with some kinetic parameters and later time points suggest that predicted parameter values should be interpreted cautiously.

When compared to previous efforts to encapsulate bendamustine, such as the non-PEGylated PLGA reported by [29], our PEGylated BR-nanoliposomes demonstrate a significant increase in encapsulation efficiency. This improvement is likely attributed to the synergistic interaction between rutin and the lipid bilayer, which appears to stabilize the vesicle structure [29].

Co-encapsulating the cytotoxic agent with rutin, a potent antioxidant, the formulation is designed to mitigate chemotherapy-induced oxidative stress and off-target systemic toxicity while utilizing the PEGylated bilayer to achieve “stealth” properties and prolonged circulation. This approach leverages the Enhanced Permeability and Retention (EPR) effect for passive tumor targeting, transforming the laboratory-scale findings into a clinically relevant strategy for sustained, targeted oncology. Furthermore, the integration of a hybrid predictive modeling framework provides a translational tool for pharmaceutical manufacturing, allowing for the precise, data-driven optimization of release kinetics to ensure consistent therapeutic performance in potential clinical applications.

### 3.9. Limitations

The current model has been designed and evaluated using four different formulations with similar lipid compositions. The future direction of study needs to be focused on expanding the range of formulations by considering different lipid proportions, PEGylation methods, and drug types.

Also, the predictive model relies on augmented datasets that were generated based on the release behavior data collected in only four experiments. Even though the reality of augmentation was validated (noise-mode mean absolute error being between 0.08% and 0.66% relative to the original values; no monotonicity violations occurring), artificial variability may not be able to capture all the uncertainties in the real world, such as variability in batches, instrumental changes, or human error.

## 4. Conclusions

Despite advances in liposomal drug delivery, current approaches remain largely formulation-specific and descriptive, with limited predictive capability. Conventional kinetic models provide empirical fits but lack generalizability, while machine learning applications are often constrained by small datasets and weak integration with mechanistic frameworks. In particular, predicting later-stage release from early experimental data remains insufficiently explored.

In this study, PEGylated nanoliposomes co-loaded with bendamustine and rutin were successfully developed and characterized, showing improved encapsulation efficiency and distinct, formulation-dependent release behavior compared to single-drug systems. Weibull modeling adequately described the nonlinear release profiles.

To address these limitations, an integrated framework combining data augmentation, monotonicity-constrained denoising, mechanistic modeling, and machine learning was implemented. In this context, it enabled prediction of later stage release within formulations (R^2^ > 0.98, MAE ≈ 0.83–1.00%). However, leave-one-formulation-out cross-validation yielded negative R^2^, thereby suggesting the need for more extensive training data in order to generalize between formulations. While curve reconstruction could be accomplished, individual parameter estimation proved unsatisfactory, owing to the compensable nature of model parameters in the Weibull model framework.

Overall, this work provides a structured and predictive framework for analyzing drug release in nanoliposomal systems, supporting more systematic formulation evaluation and offering practical potential for accelerating formulation optimization.

## Figures and Tables

**Table 1:** Particle size, polydispersity index (PDI), and zeta potential of blank, rutin-loaded, bendamustine-loaded, and co-loaded nanoliposomal formulations (mean ± SD).

Formulation	Size (nm)	PDI	Charge (mV)
Blank Nanoliposome	106.67 ± 0.61	0.108 ± 0.09	−12.47 ± 0.21
Rutin-loaded	118.30 ± 0.00	0.171 ± 0.00	−21.03 ± 0.90
Bendamustine-loaded	102.45 ± 4.91	0.167 ± 0.03	−13.33 ± 2.12
Co-loaded Formulation	99.71 ± 4.60	0.202 ± 0.01	−17.53 ± 6.75

**Table 2:** Weibull kinetic parameters (median [IQR]) estimated from replica-like augmented curves with Q∞ fixed to the 72 h release. (t_50_ denotes the time to reach 50% of Q∞ (formulation-specific asymptotic release), not 50% absolute cumulative release).

Formulation	$Q_{\infty }$ (72 h Release) %	Weibull Alpha (h)	Weibull Beta	Weibull t_50_ (h)	Weibull R^2^
BR (Bendamustine-Rutin co-loaded)	33.58 [32.61, 34.59]	13.79 [12.48, 15.26]	0.45 [0.43, 0.46]	6.00 [5.49, 6.69]	0.90 [0.88, 0.91]
RB (Rutin-Bendamustine co-loaded)	33.68 [32.75, 34.44]	2.99 [2.74, 3.26]	1.03 [0.97, 1.10]	2.09 [1.96, 2.23]	0.90 [0.86, 0.92]
B (Bendamustine-loaded)	43.02 [42.05, 44.11]	2.66 [2.45, 2.82]	0.90 [0.84, 0.95]	1.75 [1.64, 1.85]	0.94 [0.92, 0.96]
R (Rutin-loaded)	13.57 [12.95, 14.21]	1.89 [1.51, 2.40]	0.73 [0.50, 0.90]	1.12 [0.96, 1.30]	0.90 [0.85, 0.94]

**Table 3:** Coefficient of determination (R^2^) for common release-kinetic models and Korsmeyer–Peppas exponent (*n*).

Formulation	Zero-Order R^2^	First-Order R^2^	Higuchi R^2^	Korsmeyer–Peppas R^2^	n*
Bendamustine-loaded	0.8654	0.8931	0.9701	0.9412	0.28
Rutin–Bendamustine co-loaded	0.6558	0.6860	0.8258	0.9234	0.35
Bendamustine–Rutin co-loaded	0.6124	0.6510	0.7932	0.9155	0.31
Rutin-loaded	0.6101	0.6185	0.7765	0.9542	0.19

n refers to the Korsmeyer–Peppas release exponent.

**Table 4:** Forecasting performance on the held-out test set (ridge regression).

Time_h	MAE	RMSE	R^2^
6	0.872	1.134	0.9850
24	0.879	1.108	0.9873
48	0.833	1.069	0.9879
72	1.003	1.283	0.9860

**Table 5:** Prediction error (MAE, RMSE) for ML-estimated Weibull parameters ($Q_{\infty },\;\alpha ,\;\beta$) and derived $t_{50}$ on the test split.

Parameter	MAE	RMSE
Q∞	14.81365	21.78505
alpha	151.5438	538.4427
beta	0.133461	0.199211
t50	45.95743	155.7118

## Data Availability

The original experimental dataset, augmented dataset, and complete analytical code (including data augmentation, isotonic denoising, kinetic fitting, ridge regression, and LOFO cross-validation scripts) are publicly available at: https://github.com/AliOlamat/Co-Loaded-PEGylated-Nanoliposomes-.git, accessed on 16 May 2026.

## Abbreviations

B	Bendamustine-loaded formulation
BF	Bright Field
BR	Bendamustine–Rutin co-loaded formulation
DLS	Dynamic Light Scattering
DSPC	Distearoylphosphatidylcholine
DSPE-PEG (2000) amine	1,2-Distearoyl-sn-glycero-3-phosphoethanolamine polyethylene glycol 2000 amine
EE	Encapsulation efficiency
HPLC	High-performance liquid chromatography
IQR	Interquartile range
MAE	Mean absolute error
ML	Machine learning
PAVA	Pool-adjacent-violators algorithm
PDI	Polydispersity index
Q∞	Asymptotic release
R	Rutin-loaded formulation
RB	Rutin–Bendamustine co-loaded formulation
RMSE	Root mean square error
R^2^	Coefficient of determination
SD	Standard deviation
STEM	Scanning transmission electron microscopy
t_50_	Time to 50% of asymptotic release
